# Photoluminescent Eu^3+^-Doped Calcium Phosphate Bone Cement and Its Mechanical Properties

**DOI:** 10.3390/ma11091610

**Published:** 2018-09-04

**Authors:** Annemarie Oesterle, Anne V. Boehm, Frank A. Müller

**Affiliations:** Otto Schott Institute of Materials Research (OSIM), Friedrich Schiller University Jena, Löbdergraben 32, 07743 Jena, Germany; annemarie.tesch@uni-jena.de (A.O.); anne.boehm@uni-jena.de (A.V.B.)

**Keywords:** bone cement, apatite, luminescence, mechanical properties

## Abstract

Calcium phosphate cements (CPC) are well-established bone replacement materials that have been used in dentistry and orthopedics for more than 25 years. The monitoring of bone cements and the associated healing processes in the human body is difficult and so far has often been achieved using cytotoxic X-ray contrast agent additives. These additives have a negative effect on the mechanical properties and setting time of the bone cement. In this paper, we present a novel approach to prepare contrastive CPC by the incorporation of luminescent Eu^3+^-doped hydroxyapatite (Eu:HAp) nanoparticles. Eu-doped CPC (Eu:CPC) exhibited enhanced mechanical properties compared to pure CPC. Furthermore, the red photoluminescence of Eu:CPC may allow the observation of CPC-related healing processes without the use of harmful ionizing radiation.

## 1. Introduction

Mammalian hard tissues, like bone and teeth, mainly consist of the biomineral hydroxyapatite (HAp, Ca_10_(PO_4_)_6_(OH)_2_) and the protein collagen [1]. Artificial HAp of high biocompatibility and bioactivity can easily be synthesized by wet chemical precipitation or sol–gel synthesis as well as by solid-state reaction [2]. Consequently, it is used as commercial implant material in biological environments, e.g., as bone cement or bioactive coating on metallic implants [3,4] as well as for surgical bone augmentation in maxillofacial, thoracolumbar, and trauma surgery [5,6,7,8]. Calcium phosphate cements (CPC) are prepared by mixing a reactive CaP raw powder with an aqueous solution [9]. Starting from α-tricalcium phosphate (α-TCP), the resulting paste mixture sets under mineralization of calcium-deficient HAp (CDHA) [10]. Numerous formulations have been investigated to adjust the degradability, setting time, and mechanical properties of CPC [3,11,12]. Nowadays, commercial products are already used in restorative surgery and dentistry for non-load-bearing applications [3,13,14]. CDHA, one possible end product of the CPC setting reaction, is the basic material of bone apatite, which is strongly substituted in its natural form by ions such as Na^+^, K^+^, Mg^2+^, HPO_4_^2−^, and CO_3_^2−^ [1]. This shows that the crystal lattice of apatite has a high tolerance to substitutions and distortions [15]. Therefore, synthetic HAp can be doped with numerous metal ions to improve, for example, its bioactivity, degradation rate, antibacterial, optical, and magnetic properties [16,17,18,19].

For the clinical application of CPC pastes, monitoring of the material with various imaging techniques during the intraoperative examination of material insertion into the body and observation of the healing processes are highly desirable. Nowadays, the imaging properties of CPC systems are mostly enhanced by X-ray contrastive radiopacifiers such as BaSO_4_, ZrO_2_, tantalum pentoxide, bismuth salicylate, or salts of alkaline earth metals [20,21,22,23,24,25,26]. These materials affect the cement setting time and often diminish the mechanical properties of the composite [21,24,25,26]. In particular, the most frequently used additives BaSO_4_ and ZrO_2_ have a negative effect on the bone remodeling cycle and lead to inflammatory reactions due to their cytotoxic properties [27]. The incorporation of alkaline earth metals like strontium (Sr^2+^) into HAp and CPC represents a common method for increasing the X-ray contrast of CPC for biomedical imaging, due to their high molar weight and biocompatibility [25,28,29]. Nevertheless, the use of X-ray contrastive bone cements increases the number of examinations with harmful ionizing radiation and the resulting radiation exposure. On the other hand, to the best of our knowledge, the use of contrastive CPCs with luminescent markers has not been reported to date.

In this paper, we show a new approach to introduce contrasting properties in CPC by incorporating luminescent Eu-doped HAp (Eu:HAp). For this purpose, wet chemical precipitated Eu:HAp was used as powder additive during the preparation of the CPC paste. The biocompatibility of Eu:HAp was already confirmed in a previous study [30,31], and, as reported in the literature, lanthanide ions are successfully used as pharmaceutical for the therapy of bone density disorders [32]. Lanthanide ions that could be released during CPC degradation are known to have a positive effect on the bone remodeling cycle by inhibiting bone resorption [32]. Furthermore, the highly crystalline Eu:HAp seeds in Eu:CPC could improve the mechanical properties of the biomaterial. The material thus utilizes the advantageous optical and mechanical properties of Eu:CPC and represents an alternative to contrastive bone cement. In the future, this material might be used for monitoring in the field of restorative surgery, employing imaging methods that use non-ionizing radiation such as photoluminescence (PL) tomography.

## 2. Materials and Methods

### 2.1. Preparation of Eu^3+^-Doped Luminescent Bone Cement

Alpha-tricalcium phosphate (α-TCP) was prepared by sintering calcium hydrogen phosphate (CaHPO_4_, Mallinckrodt-Baker, Griesheim, Germany) and calcium carbonate (CaCO_3_, Merck, Darmstadt, Germany) in a molar ratio of 2:1 for 5 h at 1400 °C followed by quenching to room temperature. The sintered cake was crushed and passed through a 125 μm sieve followed by ball milling at 200 rpm for 4 h [33]. Eu^3+^-doped HAp (Eu:HAp) with dopant concentrations of 0.5, 1, 1.5, 2, 5, and 10 mol% (denoted as 0.5Eu:Hap, 10Eu:Hap, etc.) relative to Ca^2+^ were synthesized via co-precipitation followed by a hydrothermal treatment [31]. For the preparation of luminescent Eu:CPC, dry α-TCP and Eu:HAp powder were mixed. Here, the Eu^3+^ bulk concentration results from a statistical distribution of the Eu:HAp nanoparticles (NPs) and Eu^3+^-ions contained in in the CPC matrix. Five Eu:CPC batches with Eu^3+^ bulk concentrations ranging from 0.5 to 2.5 mol% were produced. Here, a variation of the Eu:HAp mass fraction was performed using Eu:HAp with incorporated Eu^3+^ dopant concentrations of $c_{Eu^{3+}}$ = 0.5, 1, 1.5, 2, 5, and 10 mol%, respectively. Hereby, a maximum of 50 wt% Eu:HAp powder was added, so that maximum bulk concentrations of half the $c_{Eu^{3+}}$ in Eu:HAp*_+_* were achievable. The nomenclature used for Eu:CPC was, e.g., 2Eu0.5b, which means Eu:CPC with an Eu^3+^ bulk concentration *B* of 0.5 mol% synthesized by fractional addition of 2Eu:HAp (Table 1). Also, 1 M trisodium citrate (Na_3_C_6_H_5_O_7_, Carl Roth, Karlsruhe, Germany) and 2.5 wt% disodium hydrogen phosphate (Na_2_HPO_4_, Carl Roth, Karlsruhe, Germany) were added to the mixture of α-TCP and Eu:HAp at a powder-to-liquid ratio (PLR) of 1.5 g∙mL^−1^ to add up to 50 wt% powder. The mixed Eu:CPC pastes were converted into silicon molds and stored at high humidity and 37 °C for setting.

### 2.2. Photoluminescence Measurement

Photoluminescence (PL) excitation and PL emission measurements at T = 300 K were performed using a 150 W Xe arc lamp (Tunable PowerArc Illuminator, OBB, Birmingham, NJ, USA) as the excitation source. For this purpose, the radiation was directed onto the surface of the samples arranged in an integrating sphere (IS236A-4, Thorlabs, Newton, NJ, USA). The emitted radiation was measured with a spectrometer (Maya2000 Pro, Ocean Optics, Largo, FL, USA) and corrected on the basis of the detector efficiency.

### 2.3. Mechanical Properties

Cement samples were prepared by mixing α-TCP with pure HAp instead of Eu:HAp, as the sizes of HAp and Eu:HAp NPs are very similar compared to the much larger micron-sized TCP particles. The HAp mass proportions used corresponded to the Eu:HAp mass proportions for CPC doping with 10Eu:HAp, which led to Eu^3+^ bulk concentrations of 0.5, 1, 1.5, 2, and 2.5 mol%. Thus, the corresponding mass fractions of HAp varied from 6 to 11, 17, 22, 27 wt%. For the mechanical tests, a PLR of 2.25 g∙mL^−1^ was used. For the initial setting, the samples were exposed to 100% humidity for 4 h and then transferred to distilled water for the final setting period of 7 days. The mechanical properties of HAp NP-modified CPC were characterized using a universal testing machine (Z020, Zwick, Ulm, Germany). In the three-point bending tests, a support span of L = 20 mm, a loading rate of 1 mm·min^−1^, and a preload of 0.1 N were applied. The compressive strength was tested using cuboid samples with dimensions of 6 mm × 6 mm × 12 mm, a loading rate of 10 mm·min^−1^, and a preload of 5 N. The measurement was stopped at a maximum deformation of 20%. The bending strength *σ*_b_ and compressive strength *σ*_c_ were calculated according to Equations (1) and (2), respectively, where *M* is the bending or compressive moment and *W* the modulus of resistance.

(1) $$\sigma_{b}=\frac{M_{b}}{W}=\frac{3FL}{2bh^{2}}$$

(2) $$\sigma_{c}=\frac{M_{c}}{W}=\frac{F}{bh}$$

Scanning Electron Microscopy (SEM, Sigma VP, Carl-Zeiss, Oberkochen, Germany) on pure cement and reinforced cements was performed after 7 days of setting and subsequent drying.

## 3. Results and Discussion

### 3.1. Photoluminescence

**Figure 1:**
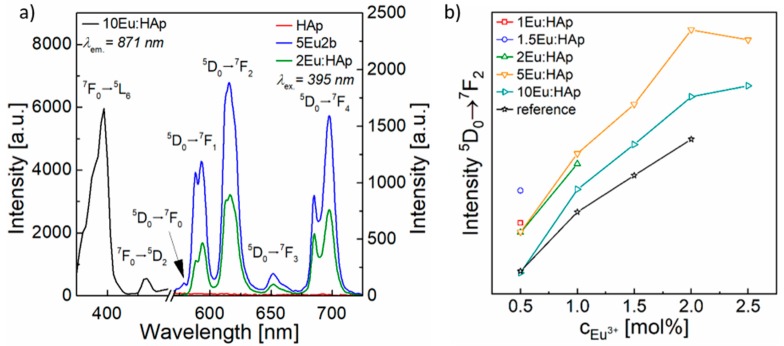
(**a**) Photoluminescence (PL) excitation of Eu:HAp and associated PL emission spectra of Eu:CPC (5Eu2b) and Eu:HAp (2Eu:HAp), both with a total concentration of Eu^3+^ *c_Eu3+_* = 2 mol%. In the case of the cement, the Eu^3+^ concentration was adjusted by adding 5Eu:HAp NPs. (**b**) Comparison of PL intensities for the dominant transition ^5^D_0_ → ^7^F_2_ in relation to the total concentration *c_Eu3+_* in all Eu:CPC produced with differently concentrated Eu:HAp NPs and the corresponding pure Eu:HAp NPs as a reference, indicating a significantly higher PL intensity for Eu:CPC than for Eu:HAp at the same Eu^3+^ bulk concentration.

The PL excitation measurements of Eu:CPC indicated the most intense absorption band for the transition ^7^F_0_ → ^5^L_6_ at 395 nm. Therefore, the excitation wavelength of *λ*_ex._ = 395 nm (Figure 1a) was selected to measure PL emission spectra. Analyses of PL emissions at T = 300 K showed the typical red emission for Eu:CPC and Eu:HAp due to the incorporation of Eu^3+^-ions. Here, five characteristic Eu^3+^ emission transitions assigned to ^5^D_0_ → ^7^F_J_ (J = 0–4) manifolds appeared around 575, 595, 613, 655, and 690 nm (Figure 1a). The most intense signals were represented by the parity forbidden electronic transitions J = 2 and 4. The manifolds J = 1 and 3 exhibited a very low intensity, and the ^5^D_0_ → ^7^F_0_ was nearly indistinguishable from the signal noise. The emission bands J = 1–4 showed Stark splitting, where each of the peaks exhibited two visible maxima caused by crystal field contributions. Because of the presence and intensity of these transition sub-manifolds, the occupation of Eu^3+^ could be assigned to Ca(I) lattice sites in HAp, as already shown in a previous study [31] and confirmed by Silva et al. [34]. Eu:HAp NPs used as a powder additive for Eu:CPC exhibited a linearly increasing PL intensity with increasing dopant concentration of up to 5 mol% Eu^3+^. At higher concentrations of Eu^3+^, the PL intensity increased much less than would be expected from linear extrapolation [31]. The PL emission intensity of Eu:CPC also increased with increasing Eu:HAp powder fraction within each Eu:CPC batch at a constant Eu^3+^ concentration in the added powder (Figure 1b). In contrast, the PL intensity of Eu:CPC with constant Eu^3+^ bulk concentration using Eu:HAp powder additives of different Eu^3+^ concentrations did not follow the already observed PL intensity trend of Eu:HAp. Here, Eu:CPC prepared with the addition of 5Eu:HAp showed the highest PL emission of all Eu:CPC, especially for high Eu^3+^ bulk concentrations. Remarkably, Eu:CPC with a constant Eu^3+^ bulk concentration exhibited a higher PL intensity than the reference Eu:HAp NPs with the same Eu^3+^ concentration (Figure 1b).

Because of the substitution of Eu^3+^ in the crystal lattice of HAp, Eu^3+^ ions were permanently bound in the HAp NP matrix, which was already shown by the XRD peak shift after incorporation of Eu^3+^ in HAp [31]. During the setting process of CPC, a basic pH milieu of pH9.5 led to the dissolution of α-TCP and subsequent precipitation of CDHA. A simultaneous dissolution of Eu:HAp NPs did not occur, as HAp is chemically stable in this pH range. Thus, there was no release of Eu^3+^ from Eu:HAp NPs and subsequent incorporation into the CPC matrix that could describe the increased PL emission. This was confirmed by the unchanged PL emission transition position and intensity ratios in the PL emission spectra. In the case of Eu:CPC, the luminescent NPs were surrounded by CPC paste. During the setting of CPC, the Eu:HAp NPs were completely encapsulated in the CDHA matrix and were thus in direct contact. In addition, the Eu:HAp NPs acted as crystallization nuclei that triggered HAp precipitation and crystal growth. Since CDHA and HAp belong to the same crystal system P6_3_/m, an epitaxial crystal growth of CDHA can be assumed from the surface of the Eu:HAp NPs. During the PL process, the radiative relaxation of the excited electrons in the PL centers of the NPs leads to visible PL. In contrast, surface defects act as electron traps that lead to non-radiative relaxation processes and reduce the PL intensity. The complete encapsulation of Eu:HAp NPs in the CDHA matrix passivated the NPs surface and led to reduced quenching losses due to a decrease in surface defect density. Therefore, the composites exhibited higher luminescence intensities than the reference powders. It is known from other host matrix systems and dopant ions that the surface quenching effects can be reduced by covering or functionalizing the NPs surface with core shell-like structures, thus healing the surface defects [35]. Energy can also migrate through the NPs and transfer excitation energy from the matrix to the luminescent Eu:HAp NPs [35]. Furthermore, the crystallite size of the single crystal Eu:HAp NPs plays an important role in surface quenching and PL intensity, as the specific surface area and associated quenching processes increase by reducing the NP size. Eu:HAp NPs showed decreasing crystallite sizes with increasing dopant concentration (D_50_(HAp) = 73 nm, D_50_(10Eu:HAp) = 55 nm). Consequently, the surface defect density of NPs increased with increasing Eu^3+^ content in Eu:HAp, due to the higher specific surface of smaller NPs. In a previous study on Eu:HAp NPs, only a slight increase in PL intensity from 5 to 10 mol% doping was observed due to concentration quenching [31]. These quenching effects of highly concentrated NPs led to an overall decrease in the luminescence of CPC.

### 3.2. Mechanical Properties

**Figure 2:**
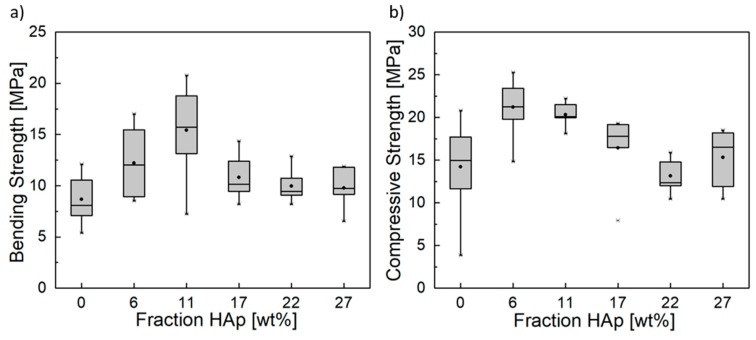
(**a**) Bending and (**b**) compressive strength of Hap-reinforced CPC.

Since wet chemically precipitated HAp NPs have a similar morphology and magnitude in crystallite size compared to Eu:HAp [31], the mechanical properties were tested with addition of HAp NP to CPC instead of Eu:HAp (Figure 2). It was found that the incorporation of moderate HAp mass fractions led to increased bending and compressive strength, while, at higher NP mass fractions, the strengths tended to values comparable to those of pure CPC (Figure 2a,b).

**Figure 3:**
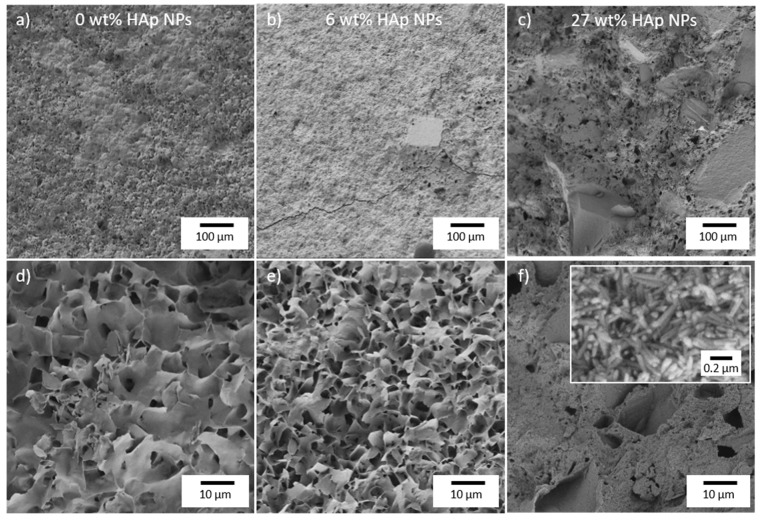
SEM micrographs of (**a**)**,** and (**d**) pure CPC, (**b**) and (**e**), CPC with a nanoparticle (NP) concentration of 6 wt%, and (**c**) and (**f**) CPC reinforced with 27 wt% HAp NPs.

HAp NPs were synthesized by wet chemical precipitation followed by hydrothermal maturation. As a result, HAp NPs exhibited a high crystallinity, especially compared to the CDHA crystals that formed during the setting reaction of CPC. Therefore, HAp NPs showed enhanced strength compared to the CDHA crystals of the CPC matrix and consequently increased the strength of the entire composite. HAp NPs sizes were in the range of 73 nm. From the literature it is known that the final CDHA micro- and nanostructure depend on the size of the raw powder used for CPC synthesis [36]. Taking this into account, HAp NPs added to the CPC paste mixture served as nuclei for the mineralization process, resulting in smaller CDHA crystals in the set CPC matrix (Figure 3). Because of the smaller CDHA crystals in CPC, interlocking of the crystals was more effective, resulting in increased bending and compressive strength with moderate addition of HAp NPs. As can be seen in the overview micrographs (Figure 3a–c), however, the ratio of unreacted powder increased with higher proportion of HAp NPs, so that a decrease in compressive strength could be observed from a HAp fraction of 17 wt%. Hereby, a higher specific surface area due to the NPs led to incomplete wetting and, thus, to partial setting.

Furthermore, a high content of HAp NPs led to a change in morphology and a decrease in the initial setting time. While the typical platelets of apatite cement were found for small fractions, needles were observed at higher NPs ratio (Figure 3f). Mixing α-TCP powder with an aqueous solution led to the dissolution of α-TCP particles up to a critical concentration from which CDHA nuclei formed and grew during cement setting. In contrast, after addition, the HAp-NPs, which were insoluble at the given pH of 9.5, acted like heterogeneous crystallization nuclei. As a result, the precipitation of CDHA from supersaturated solutions was accelerated. This led initially to smaller crystals and finally to a changed morphology of the composite. These observations suggested an epitaxial growth, which was also confirmed by the more intense luminescence due to a reduced amount of surface defects.

## 4. Conclusions

The incorporation of Eu:HAp NPs into CPC led to a composite bone cement with red light emission. Furthermore, the incorporated Eu:HAp NPs acted as nucleation seeds in the CPC paste, which led to improved interlocking of the cement matrix crystals and thus to enhanced bending and compressive strength of the material. The combination of biocompatible CPC bone cement and luminescent NPs as well as the enhanced mechanical properties make the composite a promising bone replacement material that enables monitoring without ionizing radiation.

## Figures and Tables

**Table 1:** Composition of Eu-doped calcium phosphate cements (Eu:CPC) for different bulk concentrations *B*. Concentration of the additive Eu^3+^-doped hydroxyapatite (Eu:HAp) is listed.

$c_{Eu^{3+}}\mathrm{in}\;\mathrm{the}\;\mathrm{Powder}\;\mathrm{Supplement}\;[\mathrm{mol}\%]$	Concentration of the Additive Eu:HAp $c_{Eu:HAp}\mathrm{in}\;\mathrm{Cement}\;[\mathrm{wt}\%]$				
	*B* = 0.5 mol%	*B* = 1 mol%	*B* = 1.5 mol%	*B* = 2 mol%	*B* = 2.5 mol%
1	49.7	-	-	-	-
1.5	33.2	-	-	-	-
2	25.1	50.1	-	-	-
5	10.5	20.8	31.1	41.2	51.3
10	5.6	11.2	16.7	22.1	27.4
